# The combination of valproic acid, all-trans retinoic acid and low-dose cytarabine as disease-stabilizing treatment in acute myeloid leukemia

**DOI:** 10.1186/1868-7083-5-13

**Published:** 2013-08-01

**Authors:** Hanne Fredly, Elisabeth Ersvær, Astrid Olsnes Kittang, Galina Tsykunova, Bjørn Tore Gjertsen, Øystein Bruserud

**Affiliations:** 1 Section for Hematology, Institute of Medicine, University of Bergen, Bergen, Norway; 2 Department of Medicine, Haukeland University Hospital, Bergen, Norway; 3 Institute of Medicine, Haukeland University Hospital, N-5021 Bergen, Norway

## Abstract

**Background:**

A large proportion of patients with acute myeloid leukemia (AML) are not fit for intensive and potentially curative therapy due to advanced age or comorbidity. Previous studies have demonstrated that a subset of these patients can benefit from disease-stabilizing therapy based on all-trans retinoic acid (ATRA) and valproic acid. Even though complete hematological remission is only achieved for exceptional patients, a relatively large subset of patients respond to this treatment with stabilization of normal peripheral blood cell counts.

**Methods:**

In this clinical study we investigated the efficiency and safety of combining (i) continuous administration of valproic acid with (ii) intermittent oral ATRA treatment (21.5 mg/m^2^ twice daily) for 14 days and low-dose cytarabine (10 mg/m^2^ daily) for 10 days administered subcutaneously. If cytarabine could not control hyperleukocytosis it was replaced by hydroxyurea or 6-mercaptopurin to keep the peripheral blood blast count below 50 × 10^9^/L.

**Results:**

The study included 36 AML patients (median age 77 years, range 48 to 90 years) unfit for conventional intensive chemotherapy; 11 patients responded to the treatment according to the myelodysplastic syndrome (MDS) response criteria and two of these responders achieved complete hematological remission. The most common response to treatment was increased and stabilized platelet counts. The responder patients had a median survival of 171 days (range 102 to > 574 days) and they could spend most of this time outside hospital, whereas the nonresponders had a median survival of 33 days (range 8 to 149 days). The valproic acid serum levels did not differ between responder and nonresponder patients and the treatment was associated with a decrease in the level of circulating regulatory T cells.

**Conclusion:**

Treatment with continuous valproic acid and intermittent ATRA plus low-dose cytarabine has a low frequency of side effects and complete hematological remission is seen for a small minority of patients. However, disease stabilization is seen for a subset of AML patients unfit for conventional intensive chemotherapy.

## Background

Acute myeloid leukemia (AML) is an aggressive malignancy that can only be cured by intensive chemotherapy and, if necessary, in combination with autologous or allogeneic stem cell transplantation [1]. However, due to an unacceptably high risk of early treatment-related mortality, the most intensive treatment is usually not possible for elderly patients and for patients with associated comorbidity [2,3]. These patients are therefore treated with either supportive therapy alone or in combination with AML-directed low-toxicity chemotherapy in an attempt to stabilize the disease [4,5].

Several previous studies have indicated that histone deacetylase (HDAC) inhibitors can induce disease control in AML [6,7]. Valproic acid is the HDAC inhibitor used in most of these studies, but butyric acid and depsipeptide seem to have similar effects [7]. Taken together, these previous studies demonstrate that HDAC inhibitors, usually administered in combination with all-trans retinoic acid (ATRA), induce disease stabilization with improvement of normal peripheral blood cell counts for a subset of AML patients. However, based on the overall results from several studies including more than 200 patients, complete hematological remissions seem very uncommon [8-15]. The duration of these responses varies, but they may last for more than a year. However, none of these previous studies included the systematic use of low-toxicity chemotherapy, even though such treatment was allowed in some studies to control hyperleukocytosis [9,10,12-14]. Another recent study also showed an effect of the combination of valproic acid and low-dose cytarabine, but these patients did not receive ATRA and the cytarabine dose was higher and given more frequent [16] than in our present study where we combined continuous valproic acid therapy with intermittent ATRA treatment and low-dose cytarabine. Our study is a single-institution study including a consecutive group of unselected and mainly older AML patients. Our results demonstrate that the treatment was well-tolerated and disease stabilization with improvement of normal peripheral blood cell counts was observed for a subset of patients.

## Methods

### Patients included in the study

We included 36 consecutive AML patients from our department from February 2008 to February 2012 (median age 77 years with variation range 48 to 90 years; 22 females and 14 males). All patients had non-M3-AML diagnosed in accordance with established WHO-criteria. All patients were included after written informed consent and the study was performed in accordance with the Helsinki declaration and approved by the local ethics committee (REK Vest 231.06). Collection of the clinical data was approved by the official Norwegian Social Science Data Service Authority (NSD17293) and the study was registered in public databases (ClinicalTrials.gov number NCT00995332 and EudraCT number 2007-2007-001995-36).

This was a phase II study, planned to include patients during a four year period and inclusion of at least 30 patients was expected. The approved protocol stated that inclusion should be continued and completed only if at least 2 of the first 10 patients responded to the treatment. Among the first 10 patients, we had one complete remission and 1 patient with stable disease of more than two month’s duration according to the myelodysplastic syndrome (MDS) response criteria. Our hypothesis was that the combination of a cytotoxic drug together with valproic acid + ATRA was feasible as an outpatient treatment for older and unfit AML patients. Our objectives were therefore to evaluate the effects of this treatment on (i) overall survival, (ii) the general clinical status and the disease status, and (iii) normal peripheral blood cell counts, including reticulated platelets and immunocompetent cells. The patients followed our regular control program; the Ethics Committee did not allow additional bone marrow aspirations, and for this reason extra bone marrow sampling during treatment was only done to confirm complete remission if the peripheral blood cell counts fulfilled the remission criteria.

### Control patients only receiving supportive treatment

Our results were compared with a group of 14 patients who received only supportive therapy but no AML-directed treatment. These patients represent all AML patients not receiving intensive chemotherapy from our institution that were registered in the Norwegian AML Registry during the years 2000 to August 2004 (median age 78.5 years with range 70 to 89 years; seven men and seven women). They all had a recent diagnosis of AML, that is, no patients with leukemic relapse were included in this group. They were all treated according to the same institutional general guidelines as the patients included in the present study, that is, (i) they did not receive any kind of AML-directed chemotherapy; (ii) growth factor treatment was not used; (iii) they were followed at our outpatient department at regular intervals in a similar way to the patients included in the study, and (iv) all routines for antibiotic therapy as well as platelet and erythrocyte transfusions were also similar to the study patients.

### Treatment of the patients

On day 1, the patients received an initial loading dose of valproic acid 5 mg/ml during 30 minutes and thereafter a continuous intravenous infusion (28 mg/kg) for the next 24 hours before oral treatment was started with 300 mg twice daily. This treatment was then continued indefinitely and the dose was adjusted to the highest tolerated dose not exceeding the upper limit of the therapeutic serum level of 300 to 600 μmol/L. Oral treatment with ATRA (21.5 mg/m^2^ twice daily) was given for 14 days from day 8 to 22 and repeated every 12^th^ week. Cytarabine (10 mg/m^2^ daily) was given subcutaneously on days 15 to 24 and was also repeated every 12^th^ week. For patients with rapidly increasing peripheral blood blast counts exceeding or expected to exceed 50 × 10^9^/L, all three drugs were started on day 1, but the doses and duration of treatment were similar to those described above.

### Response criteria

The international working group in AML [17] defined complete remission (CR) of AML as (i) less than 5% blasts in the bone marrow, no Auer rods and no persistence of extra-medullary disease, and (ii) neutrophil counts above 1.0 × 10^9^/L, platelet levels above 100 × 10^9^/L and transfusion independence. There is no requirement in terms of duration of this response. The MDS response criteria [18,19] generally require a duration of eight weeks for the response. The requirements for CR in MDS are (i) less than 5% blasts in the bone marrow and no dysplasia, (ii) hemoglobin level > 11 g/100 ml, neutrophil counts > 1.5 × 10^9^/L, platelet counts > 100 × 10^9^/L and (iii) no circulating blasts. These MDS criteria define stable disease as no evidence of progression for at least eight weeks.

### Estimation of reticulated platelets, Th17 and Treg cells in peripheral blood

Estimating the fraction of reticulated platelets in peripheral blood was performed as described in detail previously [20]. Frequencies of Treg and Th17 cells were also determined as described in detail previously [21].

### Statistical analyses

For comparisons between groups, we used the Mann–Whitney *U*-test; for paired observations the Wilcoxon’s signed rank test was used; and for analyses of categorized data we used the Pearson’s chi-square test. Differences were generally regarded as statistically significant when *P*-values were < 0.05.

## Results

### Patients and treatment

We included 36 patients; their clinical and biological characteristics are summarized in Table 1. During this period, five additional AML patients unfit for intensive chemotherapy were admitted to our hospital but were not included in the study; these patients were (i) one patient with relapsed AML who did not accept inclusion; (ii) two patients could not give informed consent; (iii) adequate follow-up was not possible for one patient; and (iv) one patient had already started treatment with hydroxyurea and could not be included in the protocol.

**Table 1:** The frequencies of relapsed and secondary acute myeloid leukemia (AML), signs of differentiation (FAB subclasses, CD34 expression) and genetic abnormalities among unselected AML patients unfit for intensive therapy

**FAB classification**	
M0/M1	0.41
M2	0.23
M4/M5	0.36
Expression of CD34 (> 20% positive cells)	0.70
Cytogenetic abnormalities	
Normal	0.50
Good	0.00
Intermediate	0.20
Adverse	0.30
Flt3- internal tandem duplication	0.40
NPM-1 mutation	0.35

Eleven patients had AML secondary to MDS; four of these had received treatment with 5-azacitidine before leukemic transformation and one of them had received intensive AML induction chemotherapy without reaching hematological remission before he was included in the present study. One patient had previous myelofibrosis and three patients previous polycythemia vera; two of these polycythemia vera patients had received hydroxyurea treatment. Two patients had previously received treatment with cytotoxic drugs due to breast cancer.

Seven patients had AML relapse after previous treatment - intensive chemotherapy (five patients), autologous (one patient) or allogeneic (one patient) stem cell transplantation. One of these patients had a relapse during ongoing consolidation therapy and three other patients had their relapse five months or less after consolidation therapy was completed.

Due to rapidly progressive disease with hyperleukocytosis, nine patients started with all three drugs from day 1. Furthermore, two patients died within two days after the start of therapy and were not included in our final analyses; three additional patients died within 14 days. The total mortality at two weeks was thus five patients and the total at four weeks was 12 out of 36 patients; most of these patients died from disease progression. ATRA treatment was started in all 36 patients (day 1, 9 patients; day 8, 27 patients) and completed for 24 of them, cytarabine treatment was started for 35 patients (day 1, 9 patients; day 15, 26 patients) and completed for 17 patients. The median ATRA and cytarabine doses for patients receiving reduced doses were 40% and 60%, respectively, of the planned dose. For these patients the treatment was reduced or ended due to progressive disease and/or increasing and severe thrombocytopenia. Thus, only 15 patients completed 14 days with ATRA and 10 days with cytarabine therapy together with continuous valproic acid treatment, and 14 of these patients received this therapy as a sequential treatment during the initial 24 days.

### Evaluation of response to treatment according to conventional AML criteria - 2 out of 36 patients achieved complete hematological remission

There is no general agreement with regard to which response criteria should be used for AML patients receiving low-toxicity or disease-stabilizing treatment [15], and we therefore evaluated our patients both with regard to the conventional criteria for patients receiving intensive AML chemotherapy [17] and also according to the MDS criteria [19]. Two out of 36 patients achieved CR (Table 2) of two and four months duration respectively and one of these patients had an adverse prognosis according to conventional risk stratification with AML secondary to MDS.

**Table 2:** Responses to treatment with valproic acid+all-trans retinoic acid (ATRA)+low-dose cytarabine; a summary of the results for the two acute myeloid leukemia (AML) patients achieving complete hematological remission (CR)

**Patient characteristics and treatment responses**	**Detailed description of disease characteristics and the clinical course**
CR1	Risk factor: AML secondary to previous primary myelodysplastic syndrome (MDS).
Female, 83 years old	Status at diagnosis: performance status 1, 35% blasts in bone marrow; pancytopenia with circulating blasts < 0.5 × 10^9^/L.
Treatment duration: 9 months	Valproic acid level: average level first 4 weeks 353 μmol/L and during the whole treatment period 326 μmol/L.
Response duration: 4 months Survival: 296 days	Peripheral blood cell counts: (i) platelet counts >100 × 10^9^/L for 4 months and > 30 × 10^9^/L for 7 months; (ii) neutrophils > 1.5 × 10^9^/L for 4 months; and (iii) no erythrocyte transfusions for 10 months and values Hb > 11 g/dl for 4 months.
CR2^a^	Risk factors: none, *de novo* AML.
Male, 71 years old	Status at diagnosis: performance status 3, > 20% blast in bone marrow judged from bone biopsy, pancytopenia with circulating blasts < 0.5 × 10^9^/L.
Treatment duration: 7 months	
Response duration: 2 months	Valproic acid level: average level first 4 weeks 419 μmol/L and during the whole treatment period 499 μmol/L.
Survival: 383 days	Peripheral blood cell counts: (i) > 100 × 10^9^/L for 3 months; (ii) neutrophils > 1.5 × 10^9^/L for 2 months; (iii) Hb > 11 g/100mL for 2.5 months with transfusion independence.

Patient identity refers to response (*CR* complete remission) according to the conventional AML criteria [17] and their consecutive number. Duration of treatment refers to the time receiving valproic acid, ATRA and low-dose cytarabine according to the protocol. The duration of responses refers to the time between the first and the last review documenting a peripheral blood cell value above the indicated level.

^a^Received later chemotherapy with hydroxyurea to control peripheral blood blast count.

### Evaluation of response to treatment according to MDS criteria - two patients showed complete remission and nine additional patients showed hematological improvement of normal peripheral blood cell counts lasting for at least eight weeks

Patients came to regular reviews that included clinical examination and blood sample analyses. Bone marrow aspiration was performed if peripheral blood samples showed an improvement that fulfilled the criteria for CR. According to the MDS response criteria [19], the responses had to last for a minimum of two months, that is, there should be no evidence of progression for at least two months. Based on the peripheral blood cell counts, nine of our evaluable patients fulfilled the criteria for hematological improvement based on normal peripheral blood cell counts (Table 3) in addition to the two complete remission patients (Table 2); that is, they showed an increase from pretreatment normal peripheral blood cell counts fulfilling the criteria for hematological improvement, and the MDS criteria for relapse or progression after hematological improvement were fulfilled after more than eight weeks. The nine patients with such disease stabilization were five females and four males with a median age of 77 years (range 66 to 85 years); the 23 non-responders were 16 females and seven males with a median age of 77 years (range 48 to 90 years).

**Table 3:** Responses to treatment with valproic acid+all-trans retinoic acid (ATRA)+low-dose cytarabine according to the myelodysplastic syndrome (MDS) criteria for hematological improvement (increased cell counts for at least eight weeks); a summary of the observations for nine acute myeloid leukemia (AML) patients fulfilling these criteria (referred to as stable disease, SD)

**Patient characteristics and treatment responses**	**Detailed description of disease characteristics and the clinical course**
SD1 - stable disease	Risk factor: previous primary MDS.
Male, 83 years	Status at diagnosis: performance status 0 to 2, 45% blasts in bone marrow; circulating blasts < 0.5 × 10^9^/L.
Duration of treatment: 3 months	Valproic acid level: 340 μmol/L.
Response duration: 2.5 months	Peripheral blood cell counts: (i) platelet counts > 15 × 10^9^/L for 2.5 months; (ii) neutrophils > 0.1 × 10^9^/L for 3 months; and (iii) erythrocyte level > 8.5 × 10^9^ for 2.5 months without transfusions.
Survival: 102 days	
SD2 - stable disease^a^	Risk factor: previous primary MDS.
Female, 77 years	Status at diagnosis: performance status 0 to 2, 25% blasts in bone marrow; circulating blasts < 0.5 × 10^9^/L.
Duration of treatment: 10 months	Valproic acid level: 324 μmol/L.
Response duration: 3.5 months	Peripheral blood cell counts: (i) platelet counts > 25 for 7 months; (ii) neutrophils > 0.2 × 10^9^ for 5 months; and (iii) erythrocyte level > 8.5 × 10^9^ for 3.5 months without transfusions.
Survival: 419 days	
SD3- stable disease	Risk factor: previous primary MDS.
Female, 85 years	Status at diagnosis: performance status 0 to 2, 70% blasts in bone marrow; circulating blasts < 0.5 × 10^9^/L
Duration of treatment: 5.5 months	Valproic acid level: 442 μmol/L.
Response duration: 2 months	Peripheral blood cell counts: (i) platelet counts > 18 × 10^9^ for 2 months; (ii) neutrophils could mostly not be reported; and (iii) erythrocyte level > 8.5 × 10^9^ for 3 months with stable transfusion level
Survival: 171 days	
SD4 – stable disease	Risk factor: none
Male, 74 years	Status at diagnosis: performance status 3 to 5, > 20% blasts in bone marrow; circulating blasts > 15 × 10^9^/L
Duration of treatment: 2 months	Valproic acid level: 70 μmol/L.
Response duration: 3 months	Peripheral blood cell counts: (i) platelet counts > 50 × 10^9^ for 3 months, > 100 for 1 month; (ii) neutrophils could mostly not be reported; and (iii) erythrocyte level > 8.5 × 10^9^ for 4 months with stable transfusion level.
Survival: 151 days	
SD5 – stable disease^a^	Risk factor: previous primary MDS, incomplete response to 5-azacitidine for AML.
Male, 66 years	
Duration of treatment: 7.5 months	Status at diagnosis: performance status 0 to 2, > 15% blasts in bone marrow when included in the study; circulating blasts < 0,5 × 10^9^/L.
Response duration: 2 months	Valproic acid level: 419 μmol/L.
Survival: 239 days	Peripheral blood cell counts: (i) platelet counts > 10 × 10^9^ for 2 months; (ii) neutrophils stable 0.1 × 10^9^/L for 3 months; and (iii) erythrocyte level > 8.5 × 10^9^ for 2 months without transfusions.
SD6 - stable disease	Risk factor: none
Female, 78 years	Status at diagnosis: performance status 0 to 2, > 20% blasts in bone marrow; circulating blasts < 0,5 × 10^9^/L.
Duration of treatment: 4.5 months	Valproic acid level: 476 μmol/L
Response duration: 2 months	Peripheral blood cell counts: (i) platelet counts > 30 × 10^9^/L for 2 months; (ii) neutrophils stable around 0.1 × 10^9^/L; and (iii) erythrocyte level > 8.5 × 10^9^/L for 3 months with stable transfusion frequency.
Survival: 147 days	
SD7 - stable disease^a^	Risk factor: previous primary MDS
Female, 72 years Duration of treatment: 1 month	
	Status at diagnosis: performance status 0 to 2, > 30% blasts in bone marrow; circulating blasts > 30 × 10^9^/L
	Valproic acid level: 478 μmol/L.
Response duration: 3 months	Peripheral blood cell counts: (i) platelet counts > 15 × 10^9^ for 3 months with stable transfusion frequency; (ii) neutrophils could not be reported; and (iii) erythrocyte level > 8.5 × 10^9^ for 3 months with stable transfusion frequency.
Survival: 132 days	
SD8- stable disease^a^	Risk factor: previous polycythemia vera, AML with del(5)
Male, 81 years	Status at diagnosis: performance status 0 to 2, 25% blasts in bone marrow; circulating blasts > 20 × 10^9^/L.
Duration of treatment: 4 months	Valproic acid level: 404 μmol/L
Response duration: 2 months	Peripheral blood cell counts: (i) Platelet counts > 30 × 10^9^ for 3 months; (ii) neutrophils > 0.2 × 10^9^ for 2 months; and (iii) erythrocyte level > 8.5 × 10^9^ for 2 months with stable transfusion frequency.
Survival: > 574 days	
SD9 – stable disease^a^	Risk factor: previous primary MDS.
Female, 77 years	Status at diagnosis: performance status 0 to 2, 30 % blasts in bone marrow; circulating blasts > 25 × 10^9^/L.
Duration of treatment: 3.5 months	Valproic acid level: 467 μmol/L
Response duration: > 2 months	Peripheral blood cell counts: (i) platelet counts > 25 × 10^9^ for > 2 months; (ii) neutrophils could not be reported; and (iii) erythrocyte level > 8.5 for 4 months with stable transfusion frequency.
Survival: > 141 days	

Patient identity refers to the response (*SD*, stable disease) according to the MDS criteria for hematological improvement [19] and a consecutive numbering. Duration of treatment refers to the time receiving valproic acid, ATRA and low-dose cytarabine according to the protocol. The valproic acid level is presented as the average serum level during the four first weeks of treatment. The duration of responses refers to the time between the first and the last review documenting a peripheral blood cell value above the indicated level.

^a^Received later chemotherapy with hydroxyurea or 5-mercaptopurin to control the blood blast count.

The peripheral blood values for the responders are presented in Table 4; increased levels are usually seen after one to two weeks [15] and the table therefore compares the lowest value during the first 14 days of treatment (and not the pretreatment values used for the evaluation of hematological improvement) and the highest value determined during treatment. Cell counts remained relatively low during the first two weeks of treatment and maximal responses were often reached after several weeks. Platelet responses were most common. Minor neutrophil responses were less common, and normal neutrophil counts during treatment were only seen for three patients (SD7-9) who also had normal counts at the time of diagnosis. Transfusion independence was only seen for three of the patients.

**Table 4:** Delayed increase in peripheral blood cell counts for acute myeloid leukemia (AML) patients with stable disease during treatment with all-trans retinoic acid (ATRA), valproic acid and low-dose cytarabine; a comparison of pretreatment/early treatment values versus maximal levels

	**Platelet**		**Neutrophil**		**Erythrocyte**
**Patient**	**Before or during the first 14 days**	**Highest value during treatment**	**Before or during the first 14 days**	**Highest value during treatment**	**Transfusion independent > two months**
SD1	12	49 (90)	0.1	0.7 (102)	yes
SD2	22	64 (273)	0.1	0.8 (77)	yes
SD3	< 5	79 (55)	0.4	0.4 (74)	-
SD4	103	302 (51)	0.5	0.2 (126)	-
SD5	19	51 (136)	< 0.1	0.3 (67)	yes
SD6	55	81 (106)	< 0.1	0.2 (112)	-
SD7	18	30 (15)	9.8	7.2 (27)	-
SD8	48	98 (40)	3.8	8.2 (473)	-
SD9	28	96 (141)	2.9	1.8 (49)	-

The values before treatment represent the lowest value during this time. The highest value represents the value registered after the first 14 days of treatment, and the parenthesis gives the day after start of treatment when this value was registered. The units for both platelet and neutrophil counts are × 10^9^ /L.

The 23 non-responders all had early disease progression during treatment; 19 of them had a survival shorter than eight weeks and three of them died within two weeks after the start of treatment. We also applied the response criteria as described by Ryningen *et al*. [15] who required a shorter response duration than two months to classify patients as responders, but those patients classified as responders according to these criteria corresponded to the group with remission/stable disease according to the MDS criteria.

Twelve patients (median age 77 years, range 48 to 86 years) died less than four weeks after initiation of treatment; nine of these patients had a poor performance status (3 to 5) at initiation of treatment, whereas the last three patients had a pretreatment performance status of 0 to 2 but showed chemoresistance with early progression and hyperleukocytosis/leukostasis.

### Time until increasing peripheral blood AML blast counts - the use of other cytotoxic drugs instead of low-dose cytarabine to control hyperleukocytosis

Low-dose cytarabine was included as part of the study treatment, but despite this, nine patients developed hyperleukocytosis that was treated with other cytoreductive drugs. Six of these patients were classified as initial responders (that is, stable disease) whereas three were non-responders. Even though some of the responders (indicated in Tables 2 and 3) received this additional treatment relatively early, they all lived for several months after this change; this was true both for patient SD7 (new treatment after one and a half months, lived for additional three months), patient SD5 (two months/five and a half months), patient SD9 (three and a half months/three months), patient SD8 (four and a half months/nineteen months), patient CR2 (seven months/six months) and patient SD2 (eleven months/three months). It should be emphasized that the indication for changing the cytotoxic treatment was increasing leukocytosis which is not included in the criteria for hematological improvement according to the MDS criteria. Thus, some of our responders received another cytoreductive therapy during their period of stabilization. In the nonresponder patients the new drug was started after less than four months (half, one and four months respectively) and they all had an overall survival of less than five months (one, two and five months respectively). Hydroxyurea was tried first in all these patients and for four patients, 6-mercaptopurin was also used because hydroxyurea was judged to be ineffective. The dosages of both these drugs were adjusted to the minimal dose to keep the peripheral blood blast counts below 50 × 10^9^/L.

The definition of stable disease according to the MDS criteria requires no evidence of progression for eight weeks. Increasing peripheral blood blast counts should be regarded as progression, and thus only eight of our nine patients (not patients SD7, see Table 3) with hematological improvement fulfilled the MDS definition of stable disease.

### Valproic acid serum levels did not differ between nonresponders and patients with stable disease

Valproic acid was used from day one and continued indefinitely. The dose was adjusted according to side effects; the highest tolerated dose was given and if possible the serum levels should then be within the therapeutic serum level of 300 to 600 μmol/L. The median serum level during the first four weeks of treatment did not differ between patients with remission/stable disease (median average level 404 μmol/L, variation range 70 to 478) and the nonresponders (median average level 351 μmol/L, range 100 to 545, Mann–Whitney *U*-test, *P* = 0.3202). The later valproic acid serum levels generally corresponded to the levels reached during the initial four weeks (data not shown).

### Evaluation of treatment with regard to survival - responder patients showed increased survival compared to nonresponder patients and patients only receiving supportive therapy

**Figure 1:**
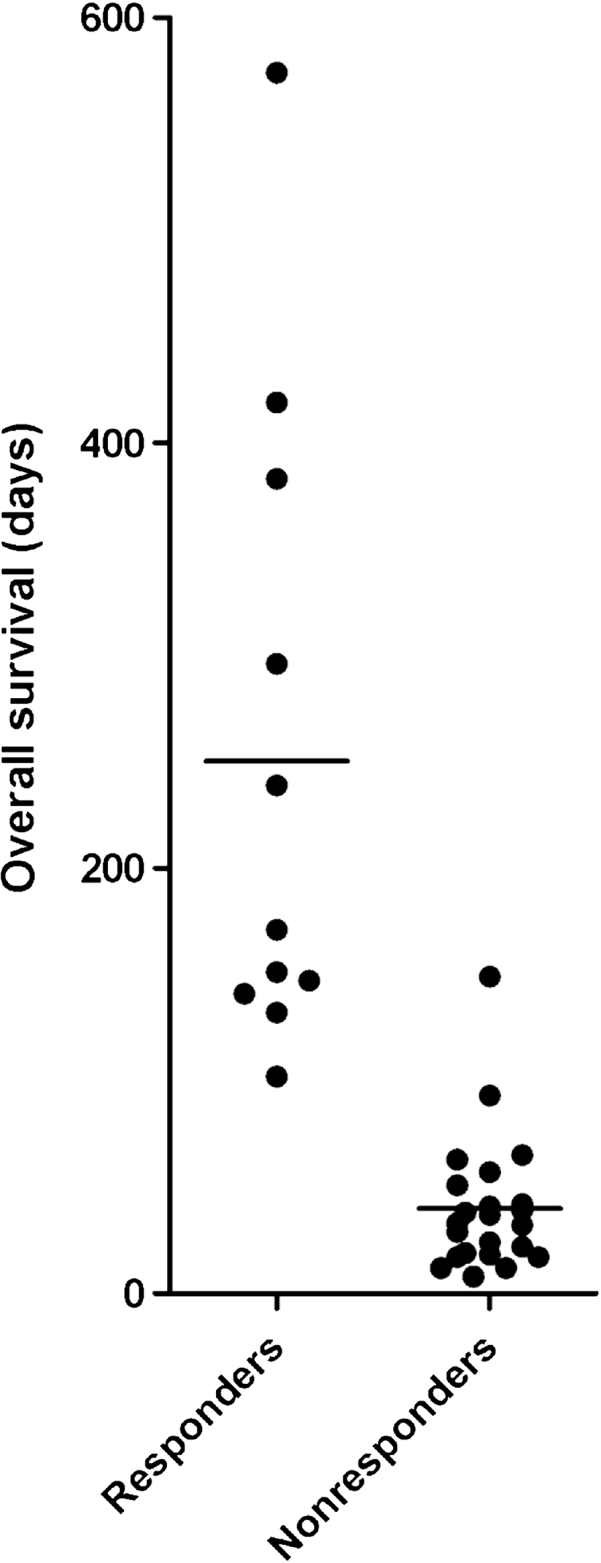
**The overall survival of acute myeloid leukemia (AML) patients after treatment with all-trans retinoic acid (ATRA), valproic acid and low-dose cytarabine.** The figure presents a comparison of responder and nonresponder patients according to the MDS criteria [19]. The mean survival is indicated for each group.

The 11 patients with complete remission and stable disease according to the MDS criteria are referred to as responders and the 23 others as nonresponders. The median survival for all our patients was 46.5 days (range 8 to >574); for the responders the median survival was 171 days (range 102 to >574) whereas the nonresponders had significantly shorter median survival of only 33 days (range 8 to 149) (Figure 1, Mann–Whitney *U*-test, *P* < 0.0001). Even our patients with stable disease but without remission showed a relatively long survival (median 151, range 102 to >574), and this was also significantly longer than for the nonresponders (Mann–Whitney *U*-test, *P* < 0.0001). Furthermore, a group of 14 control patients with newly diagnosed AML and unfit for intensive chemotherapy had a median survival of 75 days (range 1 to 215 days) and this is also significantly shorter than the nine stable disease patients and the 11 responders (*P* = 0.0140 and *P* = 0.0037 respectively, Mann–Whitney *U*-test).

There were no significant differences with regard to age, gender distribution, peripheral blood counts of leukocytes/blasts, circulating platelet or hemoglobin levels, *de novo* versus secondary leukemia, cytogenetic or molecular genetic (Flt3 or NPM1 mutations) abnormalities between responders and nonresponders in our study (data not shown). All patients had > 20% blasts in the bone marrow at the time of first AML diagnosis, as required by the WHO criteria; at the time of inclusion, three of the relapse patients had < 20% of blasts in the marrow, but for the patients included at the time of diagnosis the median blast count was 50% (range 20 to 95%) and the counts did not differ between responders and nonresponders.

The pretreatment clinical status was evaluated by the WHO performance status and by the hematopoietic cell transplantation comorbidity index (HCT-CI) [22] that has been shown to predict early death in AML patients older than 60 years of age and receiving intensive induction treatment [23]. At the start of treatment, 21 patients had a WHO performance-status between 0 (asymptomatic) and 2 (symptomatic but less than 50% in bed during the day) [24], whereas 15 patients had performance-status equal to or higher than 3 (more than 50% in bed during the day). We compared the performance status for nonresponders and responders, and found no significant difference between the two groups (*P* = 0.1193, Mann–Whitney *U*-test). Furthermore, 17 patients had a HCT-CI score of 0, corresponding to a low comorbidity risk, a scoring of 1 or 2 was seen for nine patients and 10 patients scored 3 or higher. When comparing the comorbidity index for nonresponders and responders, we did not find any statistically significant difference (*P* = 0.5256, Mann–Whitney *U*-test).

### The effect of treatment on quality of life - responder patients spend most of their time at home

Quality of life is usually reduced in AML patients at the time of diagnosis but will later stabilize [25,26]. Several instruments have been used for such analyses in randomized studies [25-27], but the aim of our nonrandomized study was to investigate whether disease stabilization in response to low-intensity treatment allowed the patients to stay at home, and we therefore investigated this endpoint by analyzing days out of hospital. Time in hospital was significantly shorter for our 11 responder patients compared with the nonresponders (Mann–Whitney *U*-test, *P* = 0.0038); the mean percentage of days in hospital relative to total survival time being 24% for the responders and 52% for the nonresponders. The median number of days in hospital was 41 days (Table 5; range 12 to 93; median survival 171 days) for the responders and 17 days (range 2 to 50; median survival 33 days) for the nonresponders.

**Table 5:** Days in hospital for acute myeloid leukemia (AML) patients responding to treatment with valproic acid, all-trans retinoic acid (ATRA) and low-dose cytarabine

**Patient**	**Overall survival (days)**	**Days in hospital relative to the total number of days:**	
		**From start of therapy to death**	**For the last 28 days before death**
CR1	296	59/ 296	0
CR2	383	42/ 383	8
SD1	102	18/ 102	14
SD2	419	20/ 419	6
SD3	171	54/ 171	4
SD4	151	93/ 151	23
SD5	239	80/ 239	18
SD6	147	35/ 147	2
SD7	132	41/ 132	4
SD8	> 574	12/ > 574	Still alive and living at home
SD9	> 141	12/ > 141	Still alive and living at home

### Treatment with valproic acid/ATRA/low-dose cytarabine reduces the levels of circulating Treg cells but does not affect Th17 levels

**Figure 2:**
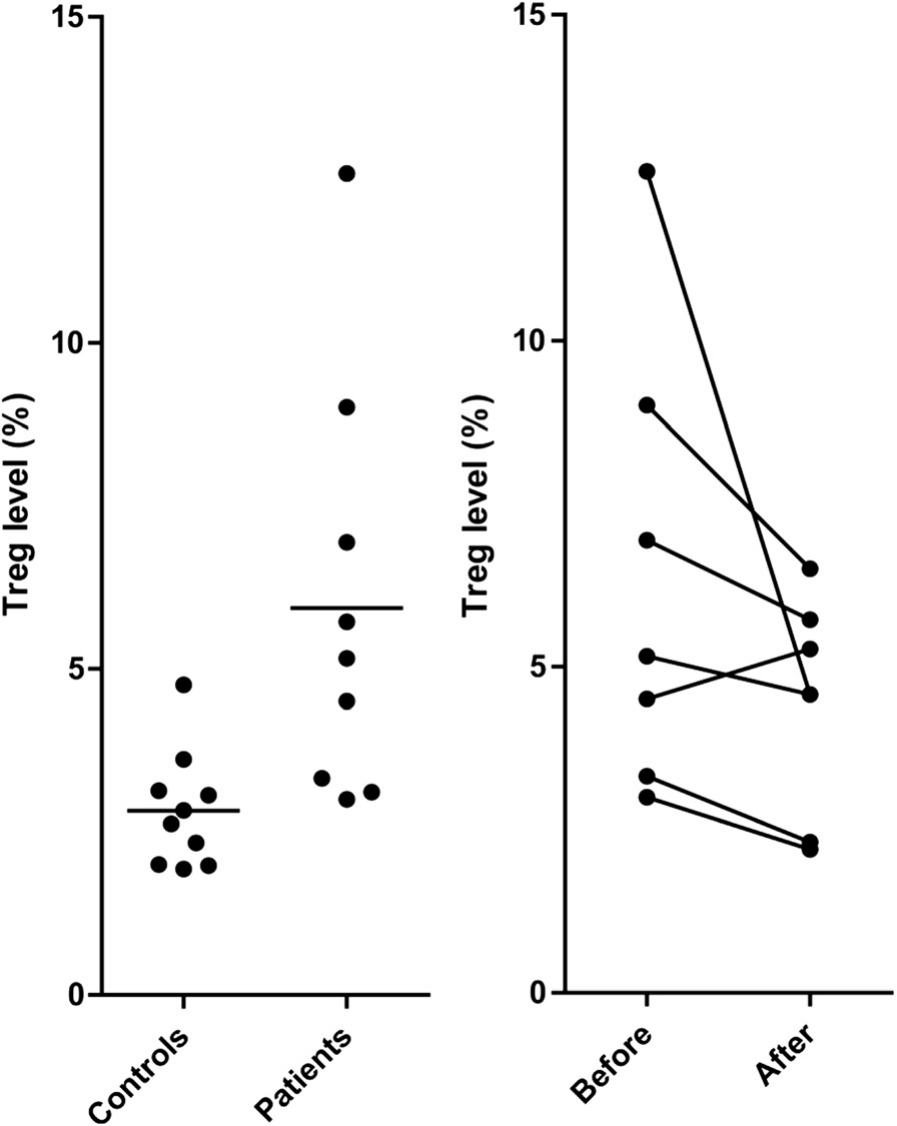
**The levels of circulating Treg cells in untreated acute myeloid leukemia (AML) and the effect of all-trans retinoic acid (ATRA), valproic acid and low-dose cytarabine on regulatory T cells (Treg) cell levels.** (Left) The levels of circulating Treg cells were determined for a consecutive group of nine AML patients and these levels were compared with a control group matched for age and gender distribution. The mean level is indicated in the figure. (Right) Treg levels were compared for seven patients before and following treatment with valproic acid, ATRA and low-dose cytarabine.

Rapid lymphoid reconstitution after AML therapy is associated with increased survival, an observation indicating that immunological events early after chemotherapy are clinically important [21,28]. Increased levels of T helper cells type 17 (Th17) and regulatory T cells (Treg) have been found in untreated AML and increased Treg levels seem to be associated with an adverse prognosis [29-31]. We therefore determined the levels of proinflammatory Th17 and Treg CD4^+^ T cells for an unselected/consecutive subset of patients included in the study. Treg levels were significantly increased in AML patients before treatment (Mann- Whitney *U*-test, *P* = 0.003) compared with healthy controls, and these levels were significantly reduced following treatment with valproic acid/ATRA/low-dose cytarabine (Figure 2; Wilcoxon signed rank test, *P* = 0.0469). For the Th17 cells, we did not observe any statistically significant differences between healthy controls, patient levels before treatment or levels following treatment with valproic acid/ATRA/cytarabine (data not shown).

### Effects of valproic acid/ATRA/low-dose cytarabine on levels of circulating reticulated platelets

Reticulated platelets represent the most recently released platelets and thereby reflect the platelet production [32]. Increasing levels after AML chemotherapy seem to predict hematopoietic reconstitution [20,32,33]. We examined the levels of reticulated platelets in patients before start of therapy (day 1), after one week of valproic acid treatment (day 8), after one additional week of combined treatment with valproic acid and ATRA (day 15), and finally after treatment with valproic acid, ATRA and 10 days of low-dose cytarabine (days 25 to 27). We detected a significant increase in reticulated platelet counts during the first 15 days of treatment (Wilcoxon’s signed rank test, *P* = 0.0106), but a similar difference could not be detected after 10 days of cytarabine treatment, and there was no difference between patients with stable disease and nonresponders (data not shown). A similar initial increase in reticulated platelets has previously been described for AML patients treated with ATRA, valproic acid and theophylline [15].

### Heat shock proteins and cytokines

We have previously examined the serum levels of heat shock protein (HSP) 70 and HSP90 as well as a wide range of cytokines for an unselected/consecutive subset of patients included in this study [34]. The cytokines included (i) immunomodulatory cytokines (CD40-Ligand, TNFα, IFNγ), (ii) interleukins (IL-1α, IL-1ß, IL-1ra, IL-2, IL-4, IL-5, IL-6, IL-10, IL-12p70, IL-13, IL-17), (iii) chemokines (CCL2/MCP-1, CCL3/MIP-1α, CCL4/MIP-1ß, CCL5/Rantes, CCL11/Eotaxin, CXCL5/ENA-78, CXCL8/IL-8, CXCL10/IP10, CXCL11/I-TAC) and (iv) the growth factors granulocyte- and granulocyte-macrophage colony-stimulating factor (G-CSF and GM-CSF), epithelial growth factor (EGF), hepatocyte growth factor (HGF), basic fibroblast growth factor (bFGF), thrombopoietin (Tpo), vascular endothelial growth factor (VEGF) and leptin. These studies were described in a previous report [34] and we now investigated whether cytokine levels showed any association with response to therapy. However, only leptin showed a significant difference and was increased in responding patients (Mann- Whitney *U*-test, *P* = 0.0033) compared to nonresponders.

## Discussion

The median age of AML patients at time of diagnosis is 65 to 70 years, and many of these patients do not receive intensive chemotherapy due to a high risk of treatment-related mortality [4,35-37]. Low-toxicity disease-stabilizing therapy based on the use of ATRA and valproic acid has a clinically relevant anti-leukemic effect for a subset of such patients even though complete hematological remissions are uncommon [8-15]. However, low-toxicity treatment with cytotoxic drugs has not been an integrated feature in any of these studies. In the present study we describe that the combination of ATRA, valproic acid and low-dose cytarabine is safe and results in disease stabilization and possibly complete hematological remission (2 out of the 36 patients) for a relatively large subset of patients.

One previous study investigated the effect of valproic acid plus low-dose cytarabine [16]. These authors also included patients with MDS; they used higher cytarabine doses that were administered more frequently and ATRA was not included. Previous studies of low-dose cytarabine have demonstrated that even this treatment can have a treatment-related mortality [38]. However, our study of an unselected patient population has demonstrated that this low-toxicity regimen can give disease stabilization for a subset of AML patients.

Response criteria for AML patients treated with intensive chemotherapy [17], and for MDS patients receiving disease-directed therapy [18,19], have been defined previously. There is no general agreement on how responses to treatment should be classified in AML patients receiving disease-stabilizing treatment. We used the MDS criteria for response evaluation in our patients, and the blast criteria for complete hematological remission are similar for AML and MDS [17-19]. The MDS criteria for stable disease require improvement/stabilization of peripheral blood cell counts for at least two months. The median survival for older AML patients not receiving disease-directed therapy is only two to three months [2,36,39], and stabilization of the disease for more than two months is therefore unexpected and should in our opinion be regarded as a response to treatment.

Our patients showed a relatively low complete remission rate of 6%; this is considerably lower than the previous MRC study that showed a remission rate of 20% after low-dose cytarabine [40]. However, the remission rate for patients receiving low-dose cytarabine seems to be dose-dependent: (i) the cytarabine dose in the MRC study was 20 mg/m^2^*twice* daily for 10 days every four to six weeks and the remission rate was then 20%; (ii) Kantarjian *et al*. [41] used cytarabine 20 mg/m^2^*once* daily for 10 days at four-week intervals and had a remission rate of 7.9%; whereas (iii) we used an even lower dose of 10 mg/m^2^ for 10 days at 12- week intervals, but despite this our remission rate of 6% is comparable to Kantarjian *et a*l. even though we included patients with AML relapse. Our present observations are also consistent with earlier AML studies of low-dose cytarabine [38].

One possible explanation for the difference in survival between our responder and nonresponder patients could be that our treatment identified a patient subset with a better prognosis. Even though a randomized clinical study is necessary to give a final answer to this question, patient selection alone seems less likely because: (i) we observed two complete remissions and spontaneous remission is very uncommon in AML; (ii) spontaneous improvement in peripheral blood cell counts is also uncommon; and (iii) many of our responders lived considerably longer (median survival of the responders > five months) than the patients in an unselected control group receiving supportive treatment according to the same institutional guidelines. However, this last comparison with historical controls should be interpreted with great care. Finally, we would emphasize that increased platelet counts and neutrophil counts > 0.5 × 10^9^/L in the responder patients (Table 4) are clinically important and decreases the risk of severe complications in these patients.

Our study had a relatively high first four-week mortality rate of 30%. Although a similar mortality has also been described in other studies of older AML patients [42], it is usually 5 to 15% in recent studies of older AML patients [41,43-45]. Menzin *et al*. [39] investigated an unselected population of 2,657 patients with newly diagnosed AML above 65 years of age; their median age was 77 years and the median survival for all patients was only two months even though 790 patients received some kind of antileukemic chemotherapy. For patients above 85 years of age, the median survival was only one month and for patients between 75 and 85 years it was two months [39]. This association between age and survival has been confirmed by others [2]. The patient population in Menzin’s study [39] is very similar to the patients included in our present study except that we also included high-risk patients with AML relapse. Thus, our relatively high early mortality can be explained by a combined effect: (i) a higher age of our patients (median 77 years, range 48 to 90 years) than for many other studies, and for such an unselected older population a very short median survival is expected [39]; (ii) many of our patients had a higher performance status than patients included in other studies and this is also associated with short survival [2,41,43-45]; (iii) our inclusion of AML relapse patients (19% of our patients) will increase the number of patients with resistant disease and thereby early progression; and (iv) there is often a delay of one to three weeks before an effect of HDAC inhibition + ATRA can be detected (Table 4). Thus, in this context, our early four-week mortality of 30% is not unexpected. In our study, cytarabine was started after 14 days of treatment with valproic acid/ATRA; our intention was to reduce the problems with cytarabine-induced cytopenia by inducing increased normal cell counts prior to this therapy. However, even though ATRA plus HDAC inhibition can reverse leukocytosis, our high early mortality may suggest that chemotherapy should start earlier when combined with ATRA/valproic acid in older unfit patients.

In a previous clinical study we described an effect of ATRA, valproic acid and theophylline on peripheral blood blast counts for a subset of AML patients [15,46]. Other studies have also described comparable results only after treatment with ATRA plus valproic acid [8-14], and for this reason we used only these two drugs in our present study and theophylline was left out. Furthermore, we showed a clinical effect when using ATRA only for two-week cycles [15]; other AML studies used ATRA as continuous therapy but we used the intermittent treatment also in our present study. This intermittent strategy of ATRA treatment is also used in AML-M3 maintenance therapy [47,48]. Finally, we added chemotherapy in our present study to increase the anti-leukemic effect and to increase the possibility of achieving better early control of hyperleukocytosis. We decided to use low-dose cytarabine because in our opinion this was the best documented low-toxicity treatment in human AML, and we also used the lowest dose previously examined in clinical trials because even low-dose cytarabine may cause treatment-related mortality (reviewed in [38]). To reduce the risk of chemotherapy-induced thrombocytopenia, we started with cytarabine after 14 days of treatment with ATRA plus valproic acid; our previous study showed that even for patients responding to this treatment with a platelet increase, there can be an initial decrease in reticulated and total platelet counts [15] and, therefore, if possible, we wanted to reduce the risk of producing a dual platelet-reducing effect.

Several HDAC inhibitors have now been developed and tried in clinical studies [49]. Vorinostat has been tried in phase I and phase II AML studies; some of these studies have combined vorinostat with demethylating agents or conventional chemotherapy, but the results for vorinostat monotherapy in human AML are comparable to the results for valproic acid treatment [5,50-53]. The same is true for MGCD0103 (mocetinostat), entinostat, panobinostat and romidepsin; to the best of our knowledge no available studies have documented that these agents are superior to valproic acid. Thus, we used valproic acid as the HDAC inhibitor in our present study because it is a safe alternative, analysis of serum levels is easily available and it seems as equally effective as other HDAC inhibitors.

During the study period, a majority of AML patients admitted to our institution and found to be unfit for intensive chemotherapy were included in our study, and several of these patients had high-risk disease (for example, high-risk cytogenetics, relapsed AML, secondary AML). Some of our stable disease/responder patients also had high-risk characteristics. Our study thus included an unselected group of patients, and responses could be seen even in high-risk patients according to conventional risk stratification [1,54].

Several previous studies of ATRA plus valproic acid-based disease-stabilizing treatments in AML have been published; these studies included a total of 244 patients and only three complete hematological remissions were then observed. Remission was thus very uncommon for these patients [9,10,12-14]. In contrast, we observed two patients with complete remission in our present study, an observation suggesting that the combination with cytotoxic drugs increases the anti-leukemic effects in ATRA plus valproic acid and makes complete remissions more common. Our present study demonstrates that low-dose cytarabine is effective and feasible, but in our opinion it is difficult to judge whether it is superior to hydroxyurea or 6-mercaptopurine. The anti-leukemic effect of low-dose cytarabine has been documented in several clinical studies [38] including a randomized study comparing this treatment with hydroxyurea [40]. However, the patients in this MRC study received higher doses of cytarabine than our patients (20 mg/m^2^ twice daily versus 10 mg/m^2^ once daily for 10 days) and the treatment was also given more frequently (every fourth to sixth week versus every third month). In addition to this, the MRC study had a majority of *de novo* AML patients (129/217 or 59%), whereas in our material only 28% had *de novo* disease. Our patients were also slightly older (median age 77 versus 74) and they had a worse WHO performance status (42% versus 30% WHO score 3 to 4). On the other hand, our present results show that oral hydroxyurea or 6-mercaptopurine could be effective for patients showing early progression after low-dose cytarabine, and the two oral alternatives are also easier to handle for the patients.

The valproic acid levels varied between patients but we could not observe any difference in the serum levels between nonresponders and responder patients. These observations strongly suggest that the valproic acid serum level is not decisive for the treatment response; a more likely explanation is that the responsiveness depends on biological characteristics of the patients.

The aim of our study was to achieve disease-stabilization/improvement so that the patients could stay at home during treatment. Our responder patients had a prolonged survival compared to the nonresponders, and most of this time was spent at home. Previous studies have shown that the quality of life is related to response to therapy [55]. In this context it seems likely that the treatment-induced AML stabilization, or even induction of remission, in our responder patients improved their quality of life.

During treatment, we detected altered levels of normal peripheral blood cells. Firstly, increased levels of reticulated platelets were detected during the initial treatment with valproic acid plus ATRA alone, and we observed a similar increase in our previous study during treatment with ATRA plus valproic acid plus theophylline [15]. Taken together, these observations suggest that theophylline is not required for these responses. Furthermore, we observed unaltered levels of pro-inflammatory Th17 cells but decreased levels of immunosuppressive Treg cells during treatment. High Treg levels are associated with an adverse prognosis in AML patients receiving intensive chemotherapy [29,31], but our present study is too small to allow any conclusion about a possible prognostic impact of Treg levels in patients receiving disease-stabilizing treatment. It is not possible to judge from our present results whether the effects of reticulated platelets, Th17 or Treg cells are directly caused by the treatment or whether they are indirect effects caused by the improved disease control.

The most important observations/conclusions from previous studies of valproic acid + ATRA treatment are summarized in Table 6[8-11,13-16,56-58], and more detailed information is given in our recent review of valproic acid in the treatment of human AML [59]. Concerning valproic acid + ATRA alone, we conclude that this is a safe treatment that induces hematological improvement for 20 to 40% of patients, but complete remissions are rare. This response rate is seen even for unselected and mainly older patients, including patients with high-risk disease. If valproic acid is combined with more intensive chemotherapy (cytarabine or 5-azacitidine) the remission rate increases, even though one exceptional and relatively small study using low-dose cytarabine, together with valproic acid and ATRA, showed no remissions among 19 patients [56]. However, it can be questioned whether the remission rate should be the only or the best marker of a clinically important effect for patients receiving disease-stabilizing antileukemic therapy [60]. This is supported by a recently published randomized study of clofarabine versus low-dose cytarabine where clofarabine increased the remission rate but did not improve overall survival [61]. In contrast, when 5-azacitidine treatment was compared with conventional care regimen (doctors’ choice of palliative care alone, low-dose cytarabine or intensive chemotherapy) the remission rates were similar, but despite this, 5-azacitidine significantly increased the overall survival even for high-risk patients [62]. It has therefore been suggested that strategies for improving survival with epigenetic therapy, without improving the remission rate, may be particularly suitable for older AML patients [60].

**Table 6:** Clinical studies of valproic acid in the treatment of human acute myeloid leukemia (AML); a summary of the previous clinical results from studies of valproic alone, valproic acid + all-trans retinoic acid (ATRA) used alone and in combination with other pharmacological agents

**Treatment**	**Studies**	**Patient number**	**Most important observations**
**Valproic acid monotherapy**	Kuendgen [12]	N = 31	This study included 31 patients receiving valproic acid alone and 40 patients receiving valproic acid plus continuous ATRA. Hematological improvement according to the myelodysplastic syndrome (MDS) criteria was also seen with valproic acid alone for a minor subset of patients.
**Valproic acid + ATRA**	Kuendgen [12]	N = 40	Based on the overall results the following conclusions can be made:
	Bellos [8]	N = 22	• This is a nontoxic treatment, the most frequent side effects being dose-dependent and thereby reversible fatigue and gastrointestinal discomfort. ATRA syndrome is very uncommon and has only been reported in one of these studies.
	Bug [9]	N = 26	
	Cimino [10]	N = 8	• The two drugs can be combined with initial low-toxicity chemotherapy to control hyperleukocytosis (hydroxyurea, cytarabine, 6-mercaptopurin).
	Raffoux [14]	N = 11	
	Pilatrino [13]	N = 20	• Complete remissions are uncommon, probably < 2 to 3%.
			• Hematological improvement as defined by the MDS criteria is seen for 20 to 40% of patients; platelet responses seem most common but erythroid and neutrophil responses may also be seen.
			• Responses can be seen even with valproic acid levels lower than the therapeutic serum level commonly used for this drug.
			• ATRA is usually used as 45 mg/m^2^/day; continuous ATRA therapy has been used in most studies but intermittent treatment for 14 days at intervals up to 12 weeks may also be used.
			• It is uncommon for responses to last for more than one year.
			• Median age in most studies is 65 to 75 years; the treatment has been used for patients up to 90 years of age.
**Valproic acid + ATRA + theophylline**	Ryningen [15]	N = 24	Continuous valproic acid + theophylline was combined with intermittent ATRA 22.5 mg/m^2^ twice daily for 15 days with 12 weeks intervals; 18% of patients had hematological improvement according to the MDS criteria, 2 patients developed atrial fibrillation and the most common side effects were dose-dependent nausea and fatigue.
**Valproic acid +ATRA + low-dose cytarabine**	Lane [56]	N = 19	Lane: no complete or partial remissions Corsetti: the study included 25 AML and 6 high-risk MDS patients; 8 patients obtained a complete remission and 3 additional patients obtained hematological improvement. Fredly: two complete remissions and 9 patients with hematological improvement.
	Corsetti [16]	N = 31	
	Fredly (present study)	N = 36	
**Valproic acid + ATRA + decitabine**	Raffoux [57]	N = 65	Raffoux: six cycles were given; 34 patients were then alive and among these 38% achieved complete remission, 6% partial remission and 41% stable disease. There were 76 events of infections.
	Soriano [58]	N = 53	
			Soriano: 42% overall response rate with 22% complete remissions. The most important nonhematological toxicity was 13 events of grade III/IV neurotoxicity.

An alternative strategy for epigenetic targeting is decitabine, another inhibitor of DNA methylation. This drug showed a complete remission rate of 24% when tested in a phase II study including only older patients (median age 74 years) [43]. A high complete remission rate was also seen in another study [63]. Decitabine has even been tried as prolonged consolidation therapy in older people [45]. Finally, an unplanned final analysis of a randomized clinical trial showed that decitabine caused a significantly increased complete remission rate, but no difference in overall survival, compared with patients’ choice after physicians’ advice (mainly low-dose cytarabine) [41]. There is therefore no general agreement on whether decitabine should be recommended for the treatment of older AML patients [64]. Rather, the observation of a high remission rate without increased overall survival supports the hypothesis by Amadori *et al*. [60] that remission rate alone may not be an optimal predictor of survival after epigenetic therapy in older AML patients.

## Conclusion

The combination of valproic acid, ATRA and low-dose cytarabine is a safe alternative for AML patients unfit for intensive chemotherapy, but only a minority of patients respond to the treatment and relatively few patients achieved complete remission. In contrast to the investigators of a previous smaller study [56] we therefore conclude that this low-toxicity therapeutic strategy should be further investigated in AML therapy, and our conclusion is also supported by a third and relatively large study that observed a relatively high complete remission rate for patients receiving this treatment [16].

## Abbreviations

AML: Acute myeloid leukemia; ATRA: All-trans retinoic acid; bFGF: Basic fibroblast growth factor; CD: Cluster of differentiation; CR: Complete remission; EGF: Epithelial growth factor; ENA-78: Epithelial-derived neutrophil-activating peptide 78; FAB: French-American-British; Flt3: Fms-like tyrosine kinase receptor 3; G-CSF: Granulocyte colony stimulating factor; GM-CSF: Granulocyte makrophage colony stimulating factor; HCT-CI: Hematopoietic cell transplantation comorbidity index; HDAC: Histone deacetylase; HGF: Hepatocyte growth factor; IFNγ: Interferon gamma; IL: Interleukin; IP-10: Interferon gamma-induced protein 10; MCP-1: Monocyte chemotactic protein-1; MDS: Myelodysplastic syndrome; MIP: Macrophage inflammatory protein; MRC: Medical Research Council; NPM-1: Nucleophosmin-1; rantes: regulatory on activation, normal T cell expressed and secreted; SD: Stable disease; Th17: T helper cells type 17; TNFα: Tumor necrosis factor alpha; Tpo: Thrombopoietin; Treg: regulatory T-cells; VEGF: Vascular endothelial growth factor; WHO: World health organization.

## Competing interests

The authors declare that they have no competing interests.

## Authors’ contributions

HF recruited patients to the study, was responsible for treatment of patients and drafted the manuscript. EE was responsible for analysis of Treg levels. AOK helped with the inclusion of patients and analysis of the data. GT helped to recruit patients to the study and to review patients during treatment. BTG contributed to the design of the study and the inclusion of patients. ØB designed the study, included and treated patients, helped with the data analyses, helped with drafting and preparing the final version of the manuscript. All authors read and approved the final manuscript.
